# Regulation of insulin-like growth factor signaling by metformin in endometrial cancer cells

**DOI:** 10.3892/ol.2014.2466

**Published:** 2014-08-20

**Authors:** YA XIE, JING-LU WANG, MEI JI, ZHONG-FU YUAN, ZHENG PENG, YI ZHANG, JIAN-GUO WEN, HUI-RONG SHI

**Affiliations:** 1 Department of Gynecology and Obstetrics, The First Affiliated Hospital of Zhengzhou University, Zhengzhou, Henan 450052, P.R. China; 2 Institute of Clinical Medicine, The First Affiliated Hospital of Zhengzhou University, Zhengzhou, Henan 450052, P.R. China

**Keywords:** metformin, insulin-like growth factor system, endometrial cancer, cell proliferation, apoptosis

## Abstract

Obesity, diabetes and insulin resistance are marked risk factors that promote the development of type I endometrial cancer. Previous studies have demonstrated that insulin-like growth factor 1 (IGF-1) and IGF-2 promote cell proliferation in endometrial cancer cells, while metformin reverses this effect and inhibits cell proliferation. However, the effects of metformin on the regulation of the IGF signaling pathway are unclear. The aim of this study was to investigate the regulation of IGF signaling by metformin in endometrial cancer cells, and to determine the effects of metformin combined with IGF-1 receptor (IGF-1R) inhibitor on cell proliferation and apoptosis. Cell proliferation was assessed following exposure of Ishikawa and HEC-1B endometrial cancer cell lines to metformin and/or the IGF-1R inhibitor, PPP. Apoptosis was assessed by TdT-mediated dUTP nick end labeling assay. Metformin was observed to downregulate IGF-1R and upregulate IGF binding protein-1 (IGFBP-1) mRNA and protein expression, while compound C, an adenosine monophosphate protein kinase inhibitor, reversed this effect. Metformin administered with PPP inhibited endometrial cancer cell proliferation to a greater degree than treatment with either agent alone. At high concentrations (1 or 2 mM), metformin induced apoptosis in endometrial cancer cells. Metformin combined with IGF-1R axis inhibitors may act synergistically to kill tumor cells, as metformin was shown to delay and prevent IGF-1R feedback. In conclusion, this study supported the results of animal studies and subclinical studies, demonstrating the feasibility of metformin combined with IGF-1R axis inhibitors in the treatment of endometrial cancer.

## Introduction

Endometrial cancer (EC) is the most frequently occurring gynecologic malignant tumor and its incidence has been increasing in recent years (1). Obesity, diabetes and insulin resistance are clear risk factors that promote the development of the more frequent type I EC (2,3). Furthermore, obesity is associated with an increased risk of EC fatality; obese women with EC have a 6.25-fold increased risk of succumbing to this disease compared with non-obese counterparts (4).

The insulin-like growth factor (IGF) system is associated with cell proliferation, obesity, diabetes and hyperinsulinemia (5). IGF signaling proteins are also important in the occurrence and development of tumors (6). Indeed, the expression levels of IGF-1, IGF-2 and IGF-1 receptor (IGF-1R) were shown to be significantly higher in EC than in the normal endometrium (7). IGF-1 and IGF-2 are mitogenic polypeptides of the IGF family and exert important roles in cell growth and differentiation. The biological actions of IGF proteins are mediated by IGF-1R, a transmembrane tyrosine kinase that is structurally associated with the insulin receptor (8–10). IGF-1R binds to the corresponding ligands, IGF-1, IGF-2 and insulin, inducing autophosphorylation. This, in turn, results in activation of distinct signaling pathways, including the phosphatidylinositol 3-kinase-AKT/mammalian target of rapamycin (mTOR) signaling pathway, eventually promoting cell proliferation and suppressing apoptosis (11).

IGF-1, IGF-2, IGF-binding protein-1 (IGFBP-1) and IGFBP-3 are expressed in normal and malignant endometrial tissues. IGFBPs bind to IGF proteins, and are involved with the regulation of cell proliferation, as well as the expression of IGFs. In the human endometrium, IGFBP-1 is the predominant IGFBP. IGFBP-1 is mainly synthesized in the liver; however, in premenopausal women, late secretory endometrial basal cells also secrete IGFBP-1. In obese and hyperinsulinemic patients, reduced levels of IGFBP-1 have been observed (12,13). Notably, the expression levels of IGF-1, IGF-2 and IGF-1R were observed to be significantly higher in stage III and IV endometrial carcinoma tissues than in stage I or II EC, and normal or hyperplastic endometrial tissue (14). In IGF-2- and IGF-1R-positive tumor cells, IGF-1R-specific repressor significantly reduced cell proliferation (14). In addition, regardless of the IGF-2 expression status, IGF-1 and IGF-1R expression levels were found to be positively correlated. These previous studies suggest that IGF-1, IGF-2 and IGF-1R expression levels are associated with the development of endometrial adenocarcinoma, highlighting the crucial role of IGF-1R function in EC and the importance of altered *IGF-1R* gene expression in the development of the malignant phenotype (15–17).

Metformin is a safe, oral, antihyperglycemic agent of the biguanides family and is widely used in the treatment of type II diabetes, particularly in obese patients. Metformin is commonly considered as an insulin sensitizer as it enhances signaling through the insulin receptor, resulting in an decrease in insulin resistance and subsequent reduction in circulating insulin levels (18). Recent studies have reported that metformin use is associated with a significant reduction in the incidence of cancer (18,19). A preliminary study suggested that metformin inhibits cancer cell growth by activating adenosine monophosphate protein kinase (AMPK), inactivating mTOR and eventually reducing the activity of the mTOR effector S6K1 (20).

In a previous study, IGF-1 and IGF-2 were demonstrated to promote EC cell proliferation, while metformin inhibited this proliferation (20). However, the effects of metformin on the IGF signaling pathway were unclear. Therefore, the aim of the present study was to investigate the regulatory mechanisms through which metformin affects the IGF signaling pathway in EC cells, and to determine the effect of metformin administered with an IGF-1R inhibitor on cell proliferation and apoptosis.

## Materials and methods

### Cell lines and reagents

The Ishikawa (IK, well-differentiated) and HEC-1B (moderately differentiated) human EC cell lines, provided by Professor LH Wei (Peking University People’s Hospital, Beijing, China), were maintained in phenol red-free Dulbecco’s modified Eagle’s medium (DMEM)/F12 with 10% fetal bovine serum (FBS) at 37°C in an atmosphere containing 5% CO_2_. The cell cultures were routinely passaged every 3–5 days. Metformin and PPP (an IGF-1R inhibitor) were purchased from Sigma-Aldrich (St. Louis, MO, USA). IGF-1 and IGF-2 were purchased from Sigma-Aldrich and R&D Systems (Minneapolis, MN), respectively. Compound C (an AMPK inhibitor) was obtained from Calbiochem (Merck Millipore, Billerica, MA, USA). Metformin was diluted in phosphate-buffered saline (PBS) as a stock solution at a concentration of 100 mM.

### Reverse transcription-quantitative polymerase chain reaction (RT-qPCR)

The IK and HEC-1B cells were plated at a density of 2×10^5^ cells/well in six-well plates for 24 h and were then treated with metformin (1, 10 or 100 μM) in the presence or absence of compound C (1 μM) in phenol red-free DMEM/F12 containing 3% steroid-stripped FBS, produced using dextran-coated charcoal (DCC-FBS) for 72 h. Total RNA was extracted from cells with TRIzol reagent (Invitrogen Life Technologies, Carlsbad, CA, USA) according to the manufacturer’s instructions. RNA was subjected to DNase I digestion to prevent possible genomic DNA contamination and then reverse-transcribed with oligo-dT primers and M-MLV Reverse Transcriptase (Promega Corporation, Madison, WI, USA). qPCR was conducted using SYBR Green sequence detection reagents (Takara Bio, Inc., Shiga, Japan) in a 20 μl reaction volume containing 1 μl cDNA, 10 μl mix, 0.4 μl Rox and 1 μl of each primer (5 μM stock). The primer sequences were as follows: IGFBP-1 forward: 5′-CTATGATGGCTCGAAGGCTC-3′; IGFBP-1 reverse: 5′-TTCTTGTTGCAGTTTGGCAG-3′; IGF-1R forward: 5′-AAGGCTGTGACCCTCACCAT-3′; IGF-1R reverse: 5′-CGATGCTGAAAGAACGTCCAA-3′; glyceraldehyde 3-phosphate dehydrogenase (GAPDH) forward: 5′-CAGTCAGCCGCATCTTCTTTT-3′, GAPDH reverse: 5′-GTGACCAGGCGCCCAATAC-3′; GAPDH forward: 5′-CTCTCTGCTCCTCCTGTTCG-3′, GAPDH reverse: 5′-TTGATTTTGGAGGGATCTCG-3′. The PCR cycling conditions were as follows: 95°C for 30 sec followed by 40 cycles of two steps at 95°C for 5 sec and 60°C for 31 sec. Fluorescent signals were detected using an ABI 7500 instrument (Applied Biosystems, Foster City, CA, USA) and the accumulation of PCR product was measured in real-time as the increase in SYBR green fluorescence. qPCR was performed in triplicate for each sample. The obtained *IGF-1R* and *IGFBP-1* mRNA levels were calculated by normalizing the threshold cycle (Ct) of *IGF-1R* and *IGFBP-1* to the Ct of *GAPDH*. The relative levels of IGF-1R and IGFBP-1 mRNA were calculated by normalizing the threshold cycle (Ct) to the Ct of GAPDH, which served as a control, using the following formula: 2-^ΔΔ^Ct. The relative levels of mRNA were then expressed as a ratio, compared with that of the control (metformin in the absence or presence of compound C).

### Western immunoblotting

The IK and HEC-1B cells were plated at a density of 2×10^5^ cells/well in six-well plates for 24 h and were then treated with metformin (1, 10 or 100 μM) in the presence or absence of compound C (1 μM) in phenol red-free DMEM/F12 containing 3% DCC-FBS for 72 h to observe the changes in IGF-1R and IGFBP-1 protein levels. Cell lysates were prepared using RIPA buffer containing 1% NP40, 0.5 sodium deoxycholate and 0.1% sodium dodecyl sulfate (SDS). Subsequently, 20 μg of each protein extract was subjected to SDS-polyacrylamide gel electrophoresis in 10% gels and subsequently electrotransferred to nitrocellulose membranes. The membranes were blocked with 5% non-fat dry milk and 0.1% Tween-20 for 1 h at room temperature (RT) with constant agitation, and then incubated with primary monoclonal rabbit anti-human GAPDH (1:1,000; Cell Signaling Technology, Danvers, MA, USA), monoclonal rabbit anti-human IGFBP1 (1:1,1000; Cell Signaling Technology) and monoclonal rabbit anti-human IGF-1R (1:1,1000; Cell Signaling Technology) antibodies overnight at 4°C. Subsequent to washing three times for 5 min each with PBS and Tween-20 (PBST), the membranes were incubated with secondary polyclonal goat anti-rabbit horseradish peroxidase-linked antibody (1:2,000; Cell Signaling Technology) for 2 h. The membranes were then washed again three times for 5 min each with PBST, and bands were visualized by enhanced chemiluminescence using the Pierce ECL Plus Western Blotting Substrates (32132, 32134) according to the manufacturer’s instructions (Pierce Biotechnology, Inc., Rockford, IL, USA). Subsequent to development, the membranes were stripped and reprobed using antibodies against GAPDH (1:1,000; Cell Signaling Technologies) to confirm equal loading. The relative protein expression levels was normalized to the GAPDH expression levels and are expressed as the ratio of treated versus untreated cells. Protein bands, including those of GAPDH, were quantified by densitometry with the Quantity One imaging program (Bio-Rad, Hercules, CA, USA).

### Cell proliferation assays

Cell proliferation assays were performed using a 5-bromodeoxyuridine (BrdU)-enzyme-linked immunosorbent assay (ELISA) kit (Roche Diagnostics GmbH, Mannheim, Germany). The IK and HEC-1B cells were plated into 96-well plates at 8×10^3^ or 1×10^4^ cells/well, respectively. At 24 h after plating, the cells were serum-starved for an additional 24 h and were then treated with increasing concentrations of metformin (0.1, 1, 10 or 100 μM) in the absence or presence of PPP (0.5 or 1 μM) for 72 h. The effects of metformin and PPP treatment were calculated as the percentage of control cell growth obtained in PBS- or DMSO-treated cells grown in the same 96-well plates. Assays were performed under serum-free conditions. DNA synthesis was monitored as determined by the incorporation of BrdU into DNA, which was detected by immunoassay according to the manufacturer’s instructions (Roche Diagnostics GmbH). Briefly, following incubation, the cells were incubated again with 10 μl/well BrdU labeling solution for an additional 2 h at 37°C. The labeling medium was removed, 200 μl/well FixDenat was added and the cells were incubated for 30 min at 20°C. Subsequently, the FixDenat solution was removed completely and the cells were incubated with 100 μl/well anti-BrdU POD working solution for 90 min at 20°C. The antibody conjugate was removed and the cells were rinsed three times with washing solution. Following removal of the washing solution, 100 μl/well substrate solution was added and the cells were incubated at 20°C for 20 min, followed by incubation with 25 μl 1 M H_2_SO_4_ for 1 min on a shaker at 100 × g. The absorbance of the samples was measured using the Fluostar Optima ELISA reader (BMG Labtech GmbH, Ortenberg, Germany) at 450 nm (reference wavelength, 690 nm). Each experiment was performed in triplicate and repeated three times to assess the consistency of the results. The BrdU assay results were compared using MTT assays and the validity of the findings was confirmed (data not shown).

### Apoptosis assay using TdT-mediated dUTP nick end labeling (TUNEL)

The apoptotic cells were detected *in situ* using a Roche TUNEL kit (Roche Diagnostics GmbH). TUNEL was conducted according to the manufacturer’s instructions to visualize the 3’-OH ends of DNA fragments in apoptotic cells. Subsequent to xylene dewaxing, the sections were rinsed three times in distilled water for 5 min and then dipped in methanol containing 0.3% H_2_O_2_ at RT for 30 min to inhibit endogenous peroxidase activity. Following rinsing in PBS three times at RT for 5 min, the sections were treated with proteinase K (Sigma-Aldrich Chemie GmbH, Mannheim, Germany) at 37°C for 8 min. The sections were then rinsed again in PBS three times at RT for 5 min, soaked in TdT buffer for 10 min and then incubated at 37°C for 60 min in a moist chamber with 50 μl TdT buffer. Subsequent to rinsing in PBS three times at RT for 5 min, the sections were placed in 50 μl fluorescein isothiocyanate (Roche Diagnostics GmbH) and then incubated at 37°C for 40 min. Following a further three 5-min rinses in PBS, the sections were dipped in 3,3′-diaminobenzidine (Roche Diagnostics GmbH) at RT for 3 min and the reaction was observed under a microscope (Olympus IMT-2; Olympus Corporation, Tokyo, Japan). The reactions were terminated with distilled water and the nuclei were counterstained with hematoxylin buffer. Normal nuclei were stained blue by DAPI, and apoptotic nuclei were stained green using TUNEL. The number of apoptotic cells was then calculated as a percentage of the total cells.

### Statistical analysis

All data are presented as the mean ± standard error of the mean. The data were analyzed by one-way analysis of variance using SPSS software (version 13.0; SPSS, Inc., Chicago, IL, USA) and P<0.05 was considered to indicate a statistically significant difference.

## Results

### Metformin downregulates IGF-1R mRNA and protein levels, and compound C reverses this effect

**Figure 1:**
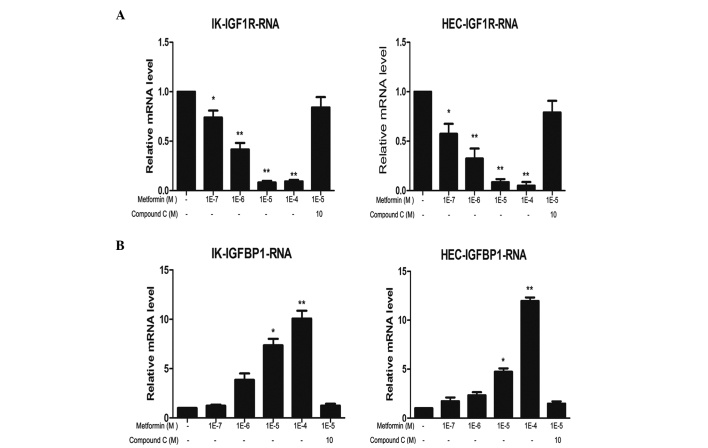
Metformin inhibits *IGF-1R* mRNA expression and promotes *IGFBP1* mRNA expression through the AMPK signaling pathway. IK (left) and HEC-1B (right) endometrial cancer cells were plated at a density of 2×10^5^ cells per well in six-well plates. After 24 h, the cells were treated with the indicated concentrations of metformin for 72 h in the presence or absence of compound C (an AMPK inhibitor). RNA was then extracted, and (A) *IGF-1R* and (B) *IGFBP-1* mRNA expression levels were quantified by reverse transcription-quantitative polymerase chain reaction (RT-qPCR). The bars indicate the mean ± standard error of the mean of three independent experiments, each performed with triplicate samples. ^*^P<0.05 vs. untreated cells and ^**^P<0.01 vs. untreated cells, by one-way analysis of variance. IGF-1R, insulin-like growth factor 1 receptor; IGFBP-1, insulin-like growth factor binding protein 1; AMPK, adenosine monophosphate protein kinase; IK, Ishikawa.

**Figure 2:**
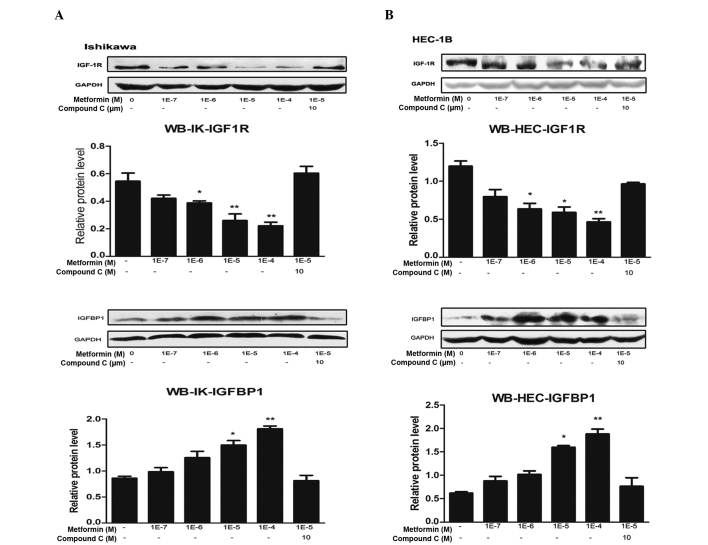
Metformin inhibits IGF-1R protein expression and promotes IGFBP-1 protein expression through the AMPK signaling pathway. (A) IK and (B) HEC-1B endometrial cancer cells were plated at a density of 2×10^5^ cells per well in six-well plates. After 24 h, the cells were treated with the indicated concentrations of metformin for 72 h in the presence or absence of compound C (an AMPK inhibitor). Protein was then extracted, and total protein was immunoblotted using specific antibodies for IGF-1R and IGFBP-1. GAPDH served as a loading control. All blots signify three independent experiments. Protein bands, including those of GAPDH, were quantified by densitometry with the Quantity One imaging program (Bio-Rad).^*^P<0.05 vs. untreated cells and ^**^P<0.01 vs. untreated cells, by one-way analysis of variance. IGF-1R, insulin-like growth factor 1 receptor; IGFBP-1, insulin-like growth factor binding protein 1; AMPK, adenosine monophosphate protein kinase; IK, Ishikawa; WB, western blotting.

The expression levels of IGF-1R mRNA and protein in IK and HEC-1B cells following treatment with metformin and/or compound C were analyzed. Metformin markedly reduced IGF-1R mRNA and protein expression levels in a concentration-dependent manner in the two cell lines. The most evident effect was observed following 100 μM metformin treatment. This inhibitory effect was partially reversed by compound C treatment (Figs. 1 and 2).

### Metformin upregulates IGFBP-1 mRNA and protein levels, and compound C reverses this effect

The effects of metformin and compound C on IGFBP-1 expression levels in IK and HEC-1B cells were analyzed. Metformin markedly increased IGFBP-1 mRNA and protein expression levels in a concentration-dependent manner in the two cell lines. Similar to IGF-1R, the most marked effect was detected following 100 μM metformin treatment. This increase was partially reversed by compound C treatment (Figs. 1 and 2).

### PPP, an IGF-1R inhibitor, suppresses cancer cell proliferation and enhances the antiproliferative effects of metformin

**Figure 3:**
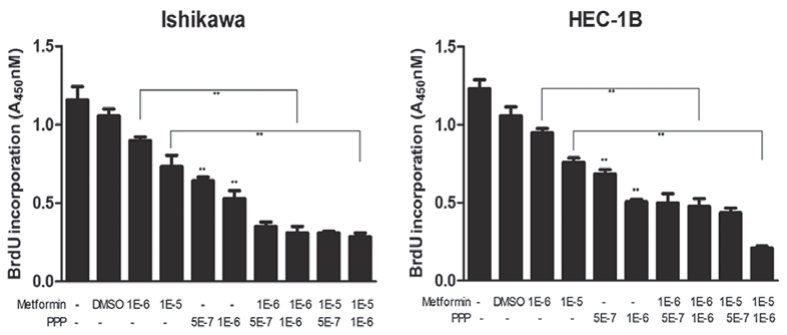
Effects of metformin and the IGF-1R inhibitor PPP on the proliferation of Ishikawa (left) and HEC-1B (right) endometrial cancer cells. The cells were serum-starved for 48 h followed by treatment with different concentrations of metformin and PPP for 72 h. Cell proliferation was then measured using the BrdU method. The results are shown as the mean ± standard error of the mean of triplicate samples, and are representative of three independent experiments. ^*^P<0.05 vs. untreated cells and ^**^P<0.01 vs. untreated cells, by one-way analysis of variance. IGF-1R, insulin-like growth factor 1 receptor; BrdU, 5-bromodeoxyuridine.

The effects of metformin with or without PPP on the proliferation of IK and HEC-1B EC cells were examined. As shown in Fig. 3, 0.5 and 1 μM PPP significantly inhibited the proliferation of EC cells (P<0.01). In addition, using BrdU incorporation assays, PPP was found to enhance the inhibitory effects of metformin on cell proliferation. The greatest effect was observed when using 1 μM PPP combined with 10 μM metformin.

### At high concentrations, metformin induces apoptosis in EC cells

**Figure 4:**
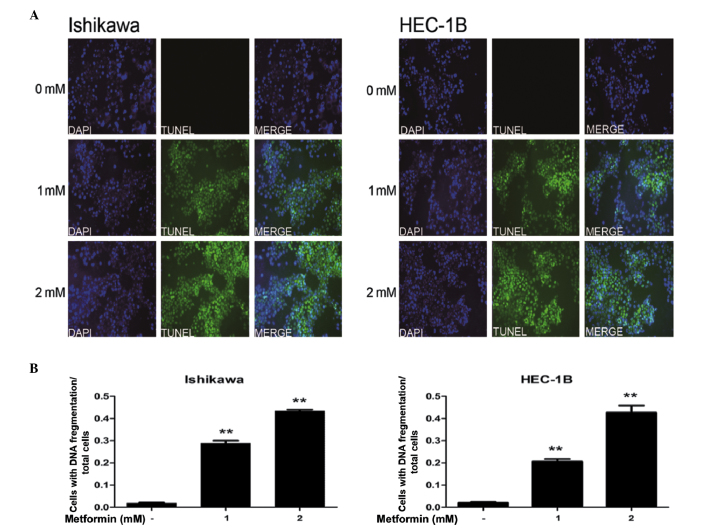
High-concentration metformin induces apoptosis. The Ishikawa and HEC-1B endometrial cancer cell lines were seeded in chamber slides. After 24 h, the cells were incubated with high concentrations of metformin for 48 h. The TUNEL method was used to detect apoptosis. ^*^P<0.05 vs. untreated cells and ^**^P<0.01 vs. untreated cells, by one-way analysis of variance. (A) DAPI (blue) staining of normal nuclei; apoptotic nuclei were stained green using TUNEL. Merge, normal nuclei and apoptotic cells. (B) Histograms show apoptotic cells as the percentage of total cells. TUNEL, terminal deoxynucleotidyl-transferase-mediated dUTP nick end labeling.

The effects of high-concentration metformin on apoptosis in EC cells were examined using TUNEL assays. The data demonstrated that incubation of IK and HEC-1B cells with 1 or 2 mM metformin significantly increased the rate of apoptosis, compared with that of the control (P<0.01; Fig. 4).

## Discussion

EC is associated with obesity and diabetes (21,22). IGF signaling proteins are expressed in endometrial tissue and are the predominant regulatory factors of steroid hormones. The IGF system has been shown to be associated with cell proliferation, obesity, diabetes, hyperinsulinemia and EC. IGF proteins and associated signaling molecules are involved in the pathogenesis of numerous types of malignant tumor, including EC (5,6,23).

IGF-1 and IGF-2 promote mitogenic signaling and exert antiapoptotic effects. Subsequent to binding to IGF-1R, IGF-1 and IGF-2 regulate IGF-1R tyrosine kinase activity to stimulate cell growth (5,20). Certain studies have observed that IGF-2 and IGF-1R are highly expressed in EC tissues compared with normal endometrial tissues (7,13,14,24). In the present study, an IGF-1R antagonist was demonstrated to inhibit the growth of EC cells. Thus, IGF-1R antagonists may be useful as secondary therapy drugs in EC patients.

Metformin significantly inhibits cell proliferation in EC cells. This effect may be associated with the increased expression levels of IGFBP1 in these cells. IGF-1 and IGF-2 promote cell proliferation in EC cells. Since IGFBP1 binds to IGFs, this reduces the levels of IGFs in the circulation. Therefore, metformin eventually reduces the levels of IGF-1 and IGF-2 in the serum, and reduces the role of IGF-1 and IGF-2 in promoting cell proliferation and inhibiting apoptosis (18,20).

The effects of metformin on apoptosis remain controversial. Cantrell *et al* (17) revealed that metformin induces apoptosis in EC, but only at high concentrations. Chen *et al* (25) demonstrated that apoptosis in metformin-treated cells was significantly higher compared with that in untreated cells, but the concentration-dependent effects of metformin on apoptosis were not observed. By contrast, Quentin *et al* (26) observed that metformin treatment does not induce apoptosis. The results of the present study are consistent with those of Cantrell *et al* (18), in that only high concentrations of metformin, not physiological concentrations, induced apoptosis in the EC cells.

Increasing clinical evidence suggests a potential correlation between biomarkers associated with the IGF1R signaling pathway and clinical benefits from IGF1R-targeted therapies. High IGF1R expression levels and elevated circulating IGF1 levels have been demonstrated to be correlated with improved response to IGF1R-targeted therapies in clinical trials of malignant tumors (27–29). In the present study, metformin combined with the IGF-1R receptor inhibitor PPP was found to markedly inhibit EC cell proliferation to a greater extent than either agent alone. This may be associated with the suppression of the IGF signaling pathway negative feedback mechanism. Metformin may be considered to simultaneously target multiple protein kinases in cancer cells, such as AMPK, S6K1, human epidermal growth factor receptor 1 (HER1), HER2 and Src (30–35). However, the majority of studies in the field have employed a simplified signal model, in which metformin functions as a general inhibitor of cancer cell growth by activating AMPK, inactivating mTOR and reducing the activity of the mTOR effector, S6K1 (36,37). Clinically, metformin may exert direct (insulin-independent) antitumor effects via inhibition of the AMPK/mTOR/S6K1 signaling pathway (37,38). However, the use of rapamycin and the corresponding analogs in the clinic has revealed that the mTOR signaling pathway is embedded in a network of signaling cross-talk and feedback mechanisms, significantly reducing the effectiveness in cancer treatment (39). If metformin has the same role as rapamycin and its analogs as inhibitor of mTOR, cancer cells may rapidly develop autoresistance to metformin-induced tumoricidal effects due to the negative feedback loop between mTORC1/S6K1 and IGF-1R/IRS-1 (40).

Therefore, IGF-1R axis inhibitors combined with metformin may act synergistically to kill tumor cells, since metformin delays and prevents feedback from the IGF-1R signaling pathway. The present study provides a theoretical foundation and new ideas which may provide a basis for further animal and subclinical studies into demonstrating the feasibility of metformin and IGF-1R axis inhibitor combination treatment in EC.
